# A conceptual model to quantify probabilistic dike breach outflow

**DOI:** 10.1007/s11069-025-07500-z

**Published:** 2025-07-21

**Authors:** Leon S. Besseling, Anouk Bomers, Jord J. Warmink, Suzanne J. M. H. Hulscher

**Affiliations:** University of Twente, Enschede, The Netherlands

**Keywords:** Flood modelling, Real-time flood forecasting, Surrogate model, Levee breach, Breach discharge, Hydrodynamic model

## Abstract

Hydrodynamic models can provide accurate information on the consequences of a dike breach, but their long computation times hinder the analysis of uncertainties and scenarios during a time-sensitive emergency situation. Conceptual models use simplified rules and relations, and allow for much faster computation while preserving reasonable accuracy. In this study, we develop a conceptual model with breach growth that estimates the dike breach outflow for varying river discharge events and for varying dike breach locations along the Rhine’s bifurcations in the Netherlands and Germany. The results show that the model is able to provide a good estimate of the breach outflow, regardless of river discharge waves shape and peak discharge. The model achieves an approximate error of 10 to 15% compared to an operational hydrodynamic model of the study area. Its computation speed allows the analysis of thousands of scenarios per minute, enabling decision makers to probabilistically analyse breach outflow hydrographs at sampled critical water levels for an incoming extreme river discharge wave. We conclude that this conceptual model can provide realistic first estimates of breach outflow for large-scale dike breaches, while requiring little input data and computational time.

## Introduction

Floods are a risk for many communities worldwide. Fluvial dike breaches can have particularly severe consequences due to the accumulation of people and economic activities in the hinterlands (Vorogushyn et al. 2011). During high river discharges, hydraulic forces can lead to dike failure in a number of ways, such as wave overtopping (Rifai et al. 2017), piping and macro-instability (Vorogushyn et al. 2009). As a breach starts to occur, water begins eroding the dike material and eventually washes away the dike. The breach also begins to grow wider, leading to a large influx of flood water into the hinterland. This breach outflow volume is the driving force of flood consequences in terms of damage and casualties (Den Heijer and Kok 2022).

Studying the impact of dike breaches is integral for guaranteeing public safety. Important management decisions such as evacuation routes and timing depend on analyses of arrival times and flood depths in the hinterland (Leskens et al. 2014; Dazzi et al. 2022). Hydrodynamic models are a common tool to obtain these insights (Teng et al. 2017). They often contain a one-dimensional (1D) river model, coupled to a two-dimensional (2D) hinterland model (e.g. Vorogushyn et al. 2011; Bomers et al. 2019; Maranzoni et al. 2022). While these models simulate river discharge and overland flooding accurately, their computation times are in the order of hours to days.

In case of an approaching extreme river discharge wave, decision makers require fast insights in the flood consequences and accompanying uncertainties for effective disaster management (Leskens et al. 2014). Uncertain factors influencing the breach outflow include (1) breach growth and final breach dimensions such as width and depth, (2) river discharge wave characteristics such as duration and peak water level, and (3) breach location (Tadesse and Fröhle 2020; Maranzoni et al. 2022). A probabilistic ensemble of model predictions and an uncertainty analysis based on the unfolding situation would allow for more informed decision making (Domeneghetti et al. 2013).

Probabilistic flood hazard analysis frameworks for dike breaches have been proposed by for example Vorogushyn et al. (2011), D’Oria et al. (2019), Curran et al. (2020) and Maranzoni et al. (2022). Often a probabilistic dike breach module (fragility curves, breach dimensions, timing, location) is coupled to a deterministic 1D unsteady river model and a 2D hinterland model. Through a Monte-Carlo simulation or repeated sampling of fragility functions, such frameworks allow a specific river discharge wave to be analysed in terms of flood hazard and breach occurrence probability. However, due to the usage of computationally demanding hydrodynamic models, these setups are too time-consuming for real-time flood modelling and disaster management. For effective support of decision-making, computation time in the order of minutes is desired (Leskens et al. 2014).

Surrogate models offer reduced computation times by implementing data-driven strategies or by leaving out characteristics of the physical system (Razavi et al. 2012). In the field of flood modelling, surrogate flood inundation models are developed that often take the outflow hydrograph as input. Bentivoglio et al. (2023) and Besseling et al. (2024) developed machine learning models for overland flow after a dike breach using the outflow hydrograph as input. Jamali et al. (2021) utilize outflow hydrograph results of a 1D sewer system model as the boundary condition of a 2D machine learning-based urban flood model. Wijaya and Yang (2021) developed a 2D flood model using cellular automata, which also requires inflow volumes to be defined as input. Obtaining the outflow hydrograph is thus an important part of modelling floods using surrogates.

Therefore, Bomers (2021) uses a data-driven surrogate model to predict the outflow hydrographs of dike breaches to a high accuracy. However, many training simulations are required to teach the data-driven model how the breach outflow develops for each location. An additional limitation mentioned by Bomers (2021) is that the neural networks were trained using only overtopping-induced breaches. Other failure mechanisms such as piping or macro-instability may lead to breaches happening when river water levels are well-below the dike crest level. Additional training simulations would be required to model these situations in data-driven models.

To overcome such limitations, others investigate the use of outflow equations based on the broad-crested (side) weir equation (e.g. Hager 1987; Lee et al. 2019). Such equations are more physically-based and through their parameters allow for varying initial conditions, such as river water levels below dike crest level. Schmitz et al. (2024) conduct a review of eleven such formulas to compute the breach outflow of a series of laboratory experiments of dike breaches. However, little attention has been paid to bridge the gap between laboratory experiments on a small scale and modelling studies of large-scale dike breaches used for disaster management planning.

In this paper we develop a simplified conceptual model to compute the outflow hydrograph of a dike breach, based on the forecasted river discharge. Conceptual models are a type of surrogate models based on modest representations of physical processes, but are much cheaper to run than conventional hydrodynamic models (Teng et al. 2017). The goal of the model developed in this paper is to quantify the effect of uncertainties, such as the moment of breaching, on the breach outflow and subsequent flood volume. This would provide decision makers with a range of possible breach outflow hydrographs and flood volumes, with which flood consequences can be probabilistically evaluated. We focus on creating the conceptual model to closely match the breach outflow output from a 1D-2D hydrodynamic model commonly used by water authorities, in part because no field observations exist in the study area.

The conceptual model utilizes existing relations and empirical equations to simulate river water levels, breach growth and breach outflow. It is a surrogate model for the deterministic 1D river model and the probabilistic breach module of other flood hazard assessment frameworks (e.g. Vorogushyn et al. 2011; Maranzoni et al. 2022), but is suitable for a fast Monte-Carlo analysis during an emergency situation. Since dike breach growth and breach outflow are strongly coupled to 2D hinterland processes, we propose this estimator of breach outflow as a first step towards a full surrogate dike breach modelling system.

This paper is structured as follows. First, the methodology explains the setup of the conceptual model, as well as the hydrodynamic model used for calibration and validation (Sect. 2). The results show the calibration on a set of river discharge waves, validation on a different set of scenarios, and the effect of the conceptual model error on subsequent flood inundation (Sect. 3). Finally, the paper ends with a discussion and a conclusion (Sects. 4 and 5, respectively).

## Methodology

### Study area

**Fig. 1:**
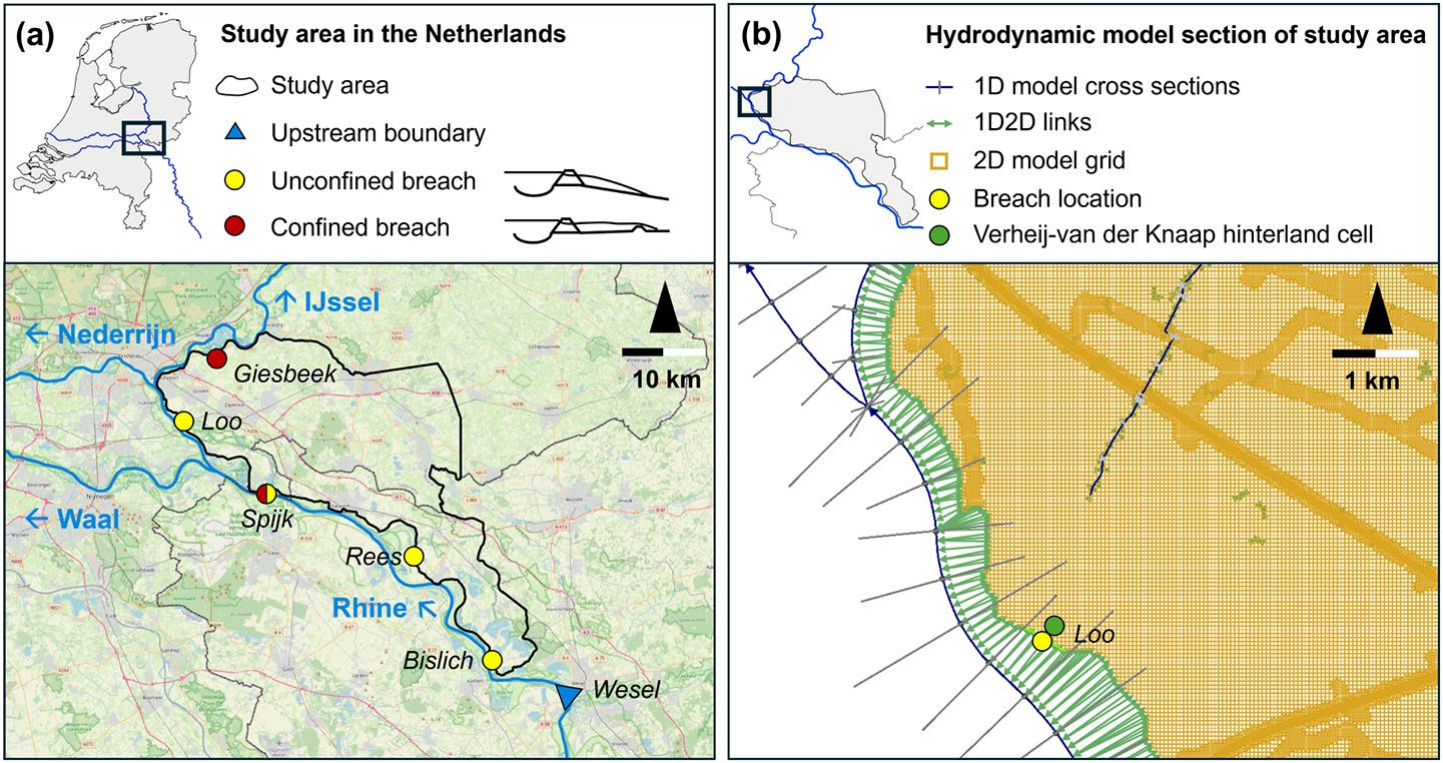
(**a**) The study area lined in black, with the five breach locations characterized as unconfined or confined breaches. The breach location at Spijk is shown as both unconfined and confined. Outflow from this breach location flows into a separate dike-ringed area, which is large enough to make it difficult to judge before the analysis if this location is best modelled as an unconfined or confined breach. (**b**) Part of the hydrodynamic model of the study area, showing the 1D river and 2D hinterland model sections

In this study, a conceptual dike breach model is set up and subsequently calibrated and validated using a numerical hydrodynamic model. The study area of this research is along the Rhine river, in the border region of the Netherlands and Germany (Fig. 1). Upon entering the Netherlands, the Rhine bifurcates into the Waal river and the Pannerdensch Canal, after which the canal bifurcates into the Nederrijn and IJssel rivers. Between the Rhine, Pannerdensch Canal and IJssel river lies an area protected by river dikes. In this study, five breaching locations along this dike-ringed area are considered (Fig. 1a). These locations are included in the flood mapping database of the local water authority, so hydrodynamic simulation data are available to calibrate and validate the conceptual model for these locations. However, the conceptual model is not limited to the application at these locations.

The breach locations can be broadly categorized in two types: (1) unconfined location with free-flow into the hinterland, and (2) partially confined locations that also exhibited submerged-flow (Devitt et al. 2023). In this paper, we will refer to these two categories as unconfined and confined, respectively. In Fig. 1a, the difference between the unconfined and confined breach types is illustrated as the degree to which the hinterland characteristics influence the breach growth and breach outflow. The general slope of the hinterland and local obstructions dictate how much the spread of the flood water is hindered, and thus how much it prevents additional river water from entering the hinterland. The distinction between unconfined and confined breaches affects the conceptual model, which is explained in Sect. 2.3.

### Hydrodynamic model

A hydrodynamic model that solves the 1D and 2D shallow water equations is used for calibration and validation of the conceptual model. The hydrodynamic model was created in the modelling software D-HYDRO (version 2023.02) by Prinsen et al. (2020) and later updated by Prinsen et al. (2022). Due to the absence of measured flood observations in the study area, this hydrodynamic model is presumed to represent the ground truth. Any errors and uncertainties in the hydrodynamic model therefore transfer to the conceptual model. However, the model was set up following the Dutch flood modelling guidelines and is currently operational for generating databases of flood scenarios at the local water authority, the water board of Rijn and IJssel. Thus, we use the hydrodynamic model’s simulated breach outflow to calibrate and validate a conceptual model for breach outflow that is considered realistic by practitioners.

The 1D part of the hydrodynamic model contains the Rhine and its bifurcations in the Netherlands (Fig. 1), and solves the complete 1D shallow water equations. The upstream boundary is located at Wesel, Germany, where a discharge wave is assigned as input. Three downstream boundaries are located approximately 100 km downstream of the study area in the Nederrijn, Waal and IJssel distributaries. The downstream boundary conditions are formed by stage-discharge (Q-h) relationships for these locations (Agtersloot and Paarlberg 2016). The computation nodes in the 1D model are 50 m apart alongside the 2D hinterland model. The 1D model extends further downstream to prevent downstream boundaries affecting the river water levels close to the breach, with the computation nodes being 100 up to 200 m apart in these downstream sections.

The 2D model mainly utilizes a cartesian computational grid cells of 40 × 40 m (Fig. 1b), solving the shallow water equations in two dimensions (depth-averaged). Nearest neighbour interpolation was used to map a high-resolution DTM (AHN3, 0.5 × 0.5 m resolution) to the coarser computational grid. In the model, raised obstacles such as roads or railroads were included as weirs with their heights taken from the high-resolution DTM. This ensures that these obstacles are not averaged away at the coarse computational grid resolution. At tunnels and underpasses, the line elements were interrupted to ensure accurate flood behaviour at these locations. Finally, the computational grid along roads or railroads was refined to 20 × 20 or 10 × 10 m to more accurately compute flow around these locations (Fig. 1b). The 2D model also contained a roughness value for each computation grid cell, based on land use classes. Finally, infiltration was included in the model via the Horton equation (Horton 1933), using uniform values for the entire study area.

Interaction between the 1D and 2D model parts occurs via 1D2D links that connect the 2D grid cells along the river to the nearest 1D cross section (Fig. 1b). These links function as weirs as soon as the river water level exceeds the dike crest level. The weir height is taken from the dikes along the rivers, using the crest levels from the high resolution DTM. Overtopping can happen at any location during the simulation at high enough river water levels, but it will not automatically trigger a breach. Breaches are initialized at a pre-defined location and moment in time, after which breach growth occurs according to the Verheij-van der Knaap (2003) equations for the lowering (Eqs. 1–2) and subsequent widening phase (Eqs. 3–5). We describe the Verheij-van der Knaap equations in detail in Sect. 2.3. The breach growth computation uses the difference between the river water level and the 2D hinterland water level at a point location specified by the modeller, chosen in this model to be about 100 m away from the breach (Fig. 1b). The breach outflow computation uses the water level difference directly across the breach.

### Conceptual model

**Fig. 2:**
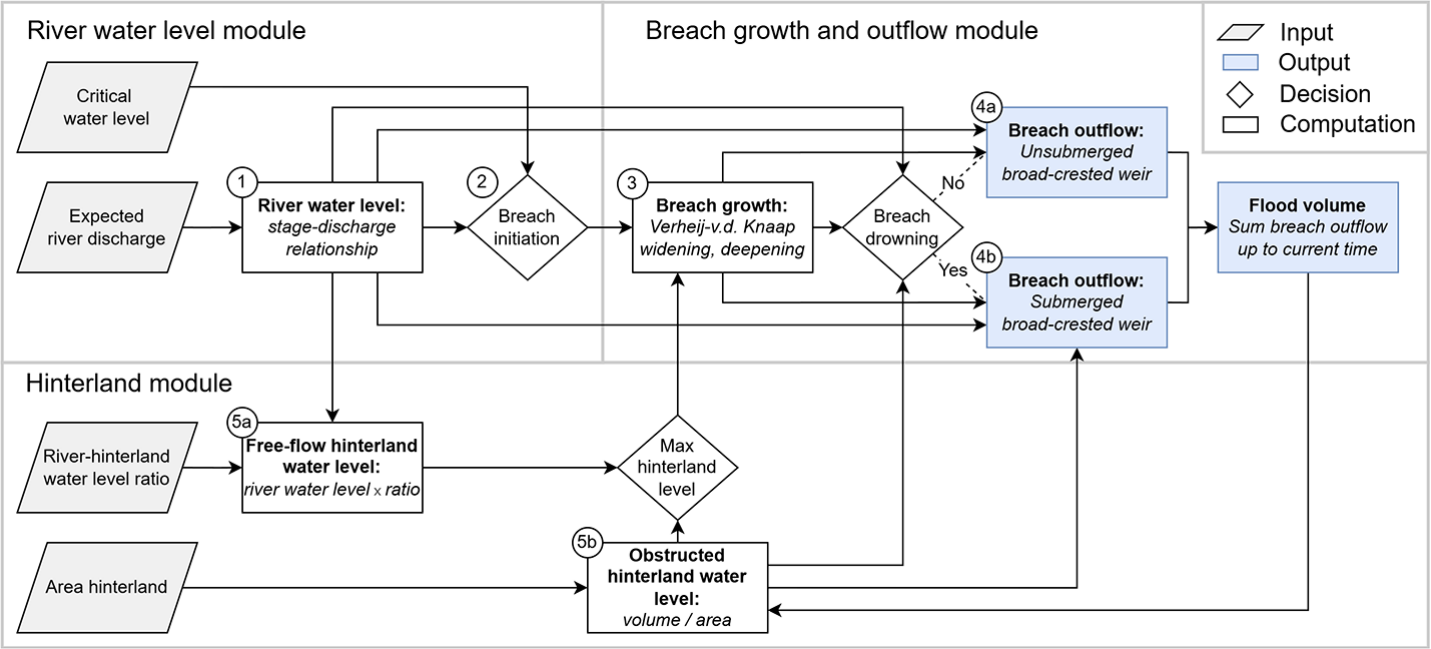
The structure of the conceptual model. For each time step of the model, the river conditions are computed. As soon as the breach is initiated, the breach starts to grow and breach outflow enters the hinterland. Depending on the area of the hinterland, the breach eventually drowns and the breach outflow is computed accordingly. Numbers at each box are for references to Fig. 2 in the main text

The boundary condition of the conceptual model is a forecasted Rhine river discharge at Lobith, just upstream of the breach location Spijk (Fig. 1). The model produces breach outflow for a particular breach location as output. The model has three key components: (1) the river water level module, (2) the breach growth and breach outflow module and (3) the hinterland water level module. A graphic representation of the model is given in Fig. 2, and the three model components and their interactions are discussed below.

#### River water level

To convert a forecasted river discharge to a river water level at the studied breach locations, we utilize local stage-discharge relations (rating curves) that were provided by the executive branch of the Dutch ministry of infrastructure and water management, Rijkswaterstaat (Fig. 2–box 1). The relations are available for every dike section along the Dutch Rhine branches, and relate the river discharge (between 6,000 and 20,000 m^3^/s) to the river water level at the breach location. The most upstream Dutch relationship is used for high discharges at the German breach locations, by increasing the computed water levels with a fixed amount. This results in water levels similar to the stage-discharge relationships of the German Federal Institute of Hydrology, which only reach up to 16,000 m^3^/s.

#### Breach initiation and growth

**Fig. 3:**
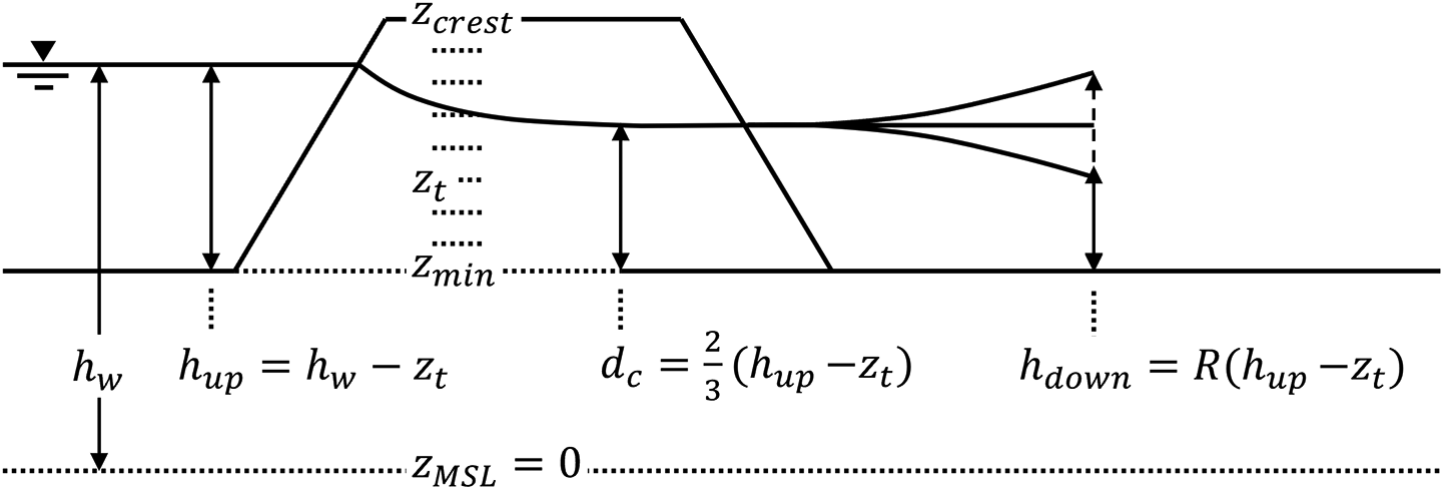
Definition sketch of the conceptual model. For each time step $\:t$, the river water level $\:{h}_{w}$ is determined with respect to the mean sea level $\:{z}_{MSL}$. The upstream water depth $\:{h}_{up}$ above the current dike crest level $\:{z}_{t}$ is computed. This is then used to compute the water depth of critical flow in the breach $\:{d}_{c}$ and the downstream water depth in the hinterland $\:{h}_{down}.$ $\:R$ is a calibration parameter explained in Sect. 2.3.3

The breach growth module is activated when the breach is initiated. Important quantities for this module are shown in the definition sketch (Fig. 3). Initiation of the breach happens when the river exceeds a threshold critical water level (Fig. 2–box 2). The time step at which this water level is reached triggers the start of the breach growth process. In the calibration and validation of the conceptual model (Sect. 2.4 and 2.5, respectively), the breach occurs when the river reaches its peak water level, to make sure this occurs at the same time step as in the hydrodynamic model. For the application of the model for uncertainty analysis, the critical water levels are sampled from fragility curves (Sect. 2.6).

Breach growth of fluvial dikes is often distinguished in two phases: an initial phase of fast breach deepening and then a main breach widening phase (Rifai et al. 2017; Goeury et al. 2022). In the conceptual model, the growth of the breach is computed using the two-phase model by Verheij-van der Knaap (Verheij 2003), to comply with the Dutch standards for flood safety and dike breach modelling. It is also included in the hydrodynamic model that is available for calibration and validation. The Verheij-van der Knaap equation is empirical and based on observations of dike breach growth. It is driven mainly by the water level difference between the river and the hinterland (Fig. 2–box 3).

During the deepening phase, the crest level of the breach decreases from the initial dike crest $\:{z}_{crest}$ to the ground level $\:{z}_{min}$ (Fig. 3). These values are dependent on the breach location. The initial breach width $\:{B}_{0}$ and the total time for the breach crest deepening $\:{t}_{d}$ have to be defined by the modeller, and are taken to be 20 m and 10 min respectively, based on the modelling procedure of the local water authority (Prinsen et al. 2020). The values are deemed realistic because of the sand-cores of the dikes in the study area, which are assumed to wash away quickly after the breach has initiated.

For $$\:t\le\:{t}_{d}$$:

1 $$\:{B}_{t}={B}_{0}$$

2 $$\:{z}_{t}={z}_{crest}-\left({z}_{crest}-{z}_{min}\right)\frac{t}{{t}_{d}}$$

Where:

- $\:{B}_{t}$ is the breach width at time $\:t$, equalling the initial breach width $\:{B}_{0}$ [m]
- $\:{z}_{t}$ is the crest level at time $\:t$, between initial crest level $\:{z}_{crest}$ and minimum crest level $\:{z}_{min}$ [m]
- $\:t$ is the time step since the breach initiation [hr]
- $\:{t}_{d}$ is the time required to erode the breach [hr]

Next, during the widening phase the breach width increases based on the water level difference between the river and hinterland $\:({h}_{up}-{h}_{down})$ at time $\:t$. The dike-specific parameters $\:{f}_{1}$ and $\:{u}_{c}$ can be chosen to reflect if the dike mainly consists of sand or more cohesive clay. Per the insight of the local water authority, we model for dikes with a sand core in this study (Prinsen et al. 2020).

For $$\:t>{t}_{d}$$:

3 $$\:{z}_{t}={z}_{min}$$

4 $$\:{B}_{t}={B}_{t-1}+\:\frac{\partial\:B}{\partial\:t}\:{\Delta\:}t$$

5 $$\:\frac{\partial\:B}{\partial\:t}=\frac{{f}_{1}{f}_{2}}{\text{l}\text{n}\left(10\right)}\frac{{\left\{g\:\left({h}_{up,\:t}-{h}_{down,\:t}\right)\right\}}^{3/2}}{{u}_{c}^{2}}\frac{1}{1+\frac{{f}_{2}\:g}{{u}_{c}}\:(t-{t}_{d})}$$

Where:

- $\:{B}_{t}$ is the breach width computed for this time step [m]
- $\:\frac{\partial\:B}{\partial\:t}$ is the change in breach width for this time step [m/h]
- $\:\varDelta\:t$ is the computational time step [hr]
- $\:{f}_{1}$ is a material factor, set to 1.2 for sand dikes (Verheij 2003).
- $\:{f}_{2}$ is a constant set to 0.04 (Verheij 2003).
- $\:g$ is acceleration due to gravity, set to 9.81 [m/s^2^].
- $\:{h}_{up,t}$ is the river water level at time $\:t$ [m]
- $\:{h}_{down,t}$ is the hinterland water level at time $\:t$ [m]
- $\:{u}_{c}$ is the critical erosion velocity, set to 0.2 m/s for sand dikes (Verheij 2003).

#### Breach type: unconfined

The hinterland water level and breach outflow processes differ slightly between unconfined breaches and confined breaches. The model distinguishes two subsequent flow phases: a free-flowing phase and a submerged or drowned phase. Unconfined breaches stay in the free-flowing phase throughout the entire simulation, because their hinterland area is infinitely large and the breach does not drown. Therefore, the hinterland water level is determined with the relationship presented in this section during the entire simulation (Fig. 2–box 5a). This hinterland water level $\:{h}_{down}$ is required for the Verheij-van der Knaap breach growth equation at every time step $\:t$, as higher hinterland water levels lead to slower breach growth.

The Verheij-van der Knaap method relies on a location in the hinterland beyond the breach to evaluate $\:{h}_{down}$. The closer to the breach this location is picked, the faster $\:{h}_{down}$ increases during the flood, and the sooner the breach growth slows down. If the location is picked further away from the breach, the breach grows more quickly. The hydrodynamic model uses a 2D hinterland grid cell 100 m beyond the breach for this location (Sect. 2.2 and Fig. 1b).

In the conceptual model, it is assumed that critical flow through the breach establishes immediately and that the breach functions as an unsubmerged broad-crested weir (Fig. 3). Therefore, the flow depth in the breach is considered the critical depth, which is 2/3 of the upstream energy head in the river (e.g. Chow 1959). We assume the river water level above the crest level to approximate the head:

6 $$\:{d}_{c}=\frac{2}{3}({h}_{up}-{z}_{t})$$

Since the conceptual model does not include a 2D component, no single point location in the hinterland can be defined to evaluate $\:{h}_{down}$. In reality, the flow of water through the breach enters the hinterland and is free to fan out across the surface. It also experiences the surface roughness, as well as effects of local elevation differences. Therefore, it is unreasonable to assume that the hinterland water level $\:{h}_{down}$ just beyond the breach exactly equals the depth of critical flow in the breach $\:{d}_{c}$. This is why we consider a calibration parameter $\:R$ in this research, to be calibrated for a breach location (Fig. 3). It represents the ratio between the river water level and the hinterland water level. $\:R$ equals 2/3 in a base scenario for unconfined breaches, and it can vary between 0 and 1 due to varying effects per breach scenario. These upper and lower bounds mean that the hinterland water level at least equals ground level $\:(R=0)$, and at most equals river water level $\:(R=1)$. Thus, a lower value of $\:R$ means a faster growing breach with higher outflow, and a higher value of $\:R$ results in a slower growing breach with lower outflow.

7 $$\:{h}_{down,t}=R\:({h}_{up,t}-{z}_{t})$$

The hinterland water level $\:{h}_{down}$ is required for the breach widening (Eq. 5). It does not affect the breach outflow for unconfined breaches, since the water is assumed to always maintain critical flow through the breach. Outflow from the breach at time $\:t$ is modelled as an unsubmerged broad-crested weir (Fig. 2–box 4a) using Eq. 8, as soon as the dike crest level is below the river water level.

8 $$\:{Q}_{br,t}=m{\left(\frac{2}{3}\right)}^{3/2}\sqrt{g}\:{B}_{t}\:{\left({h}_{up,t}-{z}_{t}\right)}^{3/2}$$

Where $\:m$ is a discharge coefficient approximating 1 for dikes that erode until the level of the surrounding ground level (Visser 1998). Similar to the hydrodynamic model, we assume this to be the case in the conceptual model.

#### Breach type: confined

For confined breaches, the conceptual model initially behaves according to the free-flowing principles. The hinterland water level is again computed using the calibration parameter $\:R$ of Eq. 7 (Fig. 2–box 5a). However, the hinterland water level will gradually increase depending on the characteristics of the study area such as hinterland slope or retention capacity. At some point, enough breach outflow has entered the hinterland for the water level $\:{h}_{down}$ to exceed the depth of critical flow through the breach $\:{d}_{c}$. The conceptual model then switches to the submerged phase of the simulation, and the hinterland water level affects the breach outflow (Fig. 2–box 4b) and the breach growth (Fig. 2–box 5b).

Equation 9 is used to conceptually model the hinterland water level in a zero-dimensional (0D) manner. The volume of the total breach outflow $\:V$ up to that point in time is divided by a calibrated parameter $\:A$ representing the area of the hinterland (Fig. 2, box 5b). This area is not correlated to the physical size of the study area, because local elevation changes or raised obstacles like (rail)roads can lead to quick breach drowning in the case of confined breaches, even while the floodable area can be quite large. Therefore, $\:A$ is a parameter that influences the rise rate of the hinterland water level and thus the time it takes for the breach to drown.

9 $$\:{h}_{down,t}=\frac{{V}_{t}}{A}$$

During the free-flowing phase of a confined breach location, breach outflow is initially modelled following Eq. 8 (Fig. 2–box 4a). However, after a while the hinterland water level causes drowning of the breach. Following literature (e.g. Nikolov et al. 1978; Visser 1998; Kamrath et al. 2006), the critical flow phase ends and a sub-critical phase starts when the hinterland water level$\:\:{h}_{down}$ has increased to be greater than the depth of critical flow in the breach $\:{d}_{c}=\frac{2}{3}{h}_{up}$ (Eq. 6). Then the submerged broad-crested weir principle (Eq. 10) is used, in which an increasing hinterland water level results in a decreasing breach outflow (Fig. 2–box 4b).

10 $$\:{Q}_{br,t}=m\:{B}_{t}\sqrt{2g\left({h}_{up,t}-{h}_{down,t}\right)}({h}_{down,t}-{z}_{t})$$

### Calibration

The conceptual model requires calibration for each breach location, because varying hinterland characteristics lead to different breach developments. Calibration is conducted with respect to hydrodynamic simulations that were conducted by the water authority responsible for the study area (Water board Rijn & IJssel) with the hydrodynamic model described in Sect. 2.2. The calibration of the conceptual model optimizes the flood volume time series, since flood volume is the driving force of damage and casualties in the hinterland (Den Heijer and Kok 2022). Initial tests with calibration on the breach outflow time series resulted in slightly worse flood volume predictions.

The available simulations utilize a standardized river discharge wave shape from the GRADE-project (Hegnauer et al. 2014). This project generated a time series of discharge waves for the Rhine of 50,000 years, using a weather generator coupled to hydrologic and hydraulic models. The average discharge wave shape is used by Dutch authorities for modelling (Table 1). The wave was scaled to correspond to return periods of 100 and 100,000 years (T100 and T100,000 in Table 1). The dikes are set to breach when the river reaches the peak water level, at the same time step in both the conceptual and hydrodynamic model.

**Table 1:**
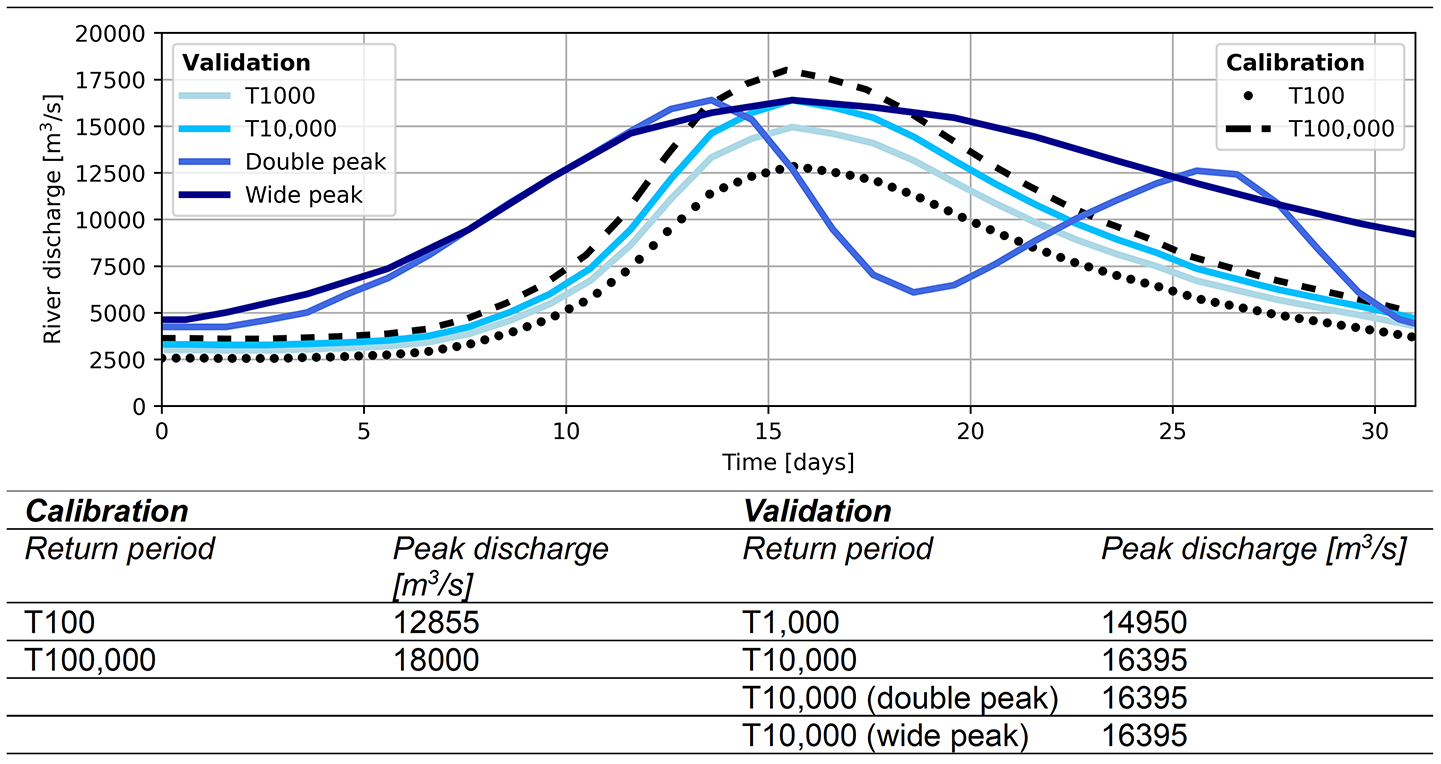
Calibration and validation discharge wave shape, return periods and peak discharges. Time simulated per discharge wave is one month

#### Unconfined breaches

For unconfined breaches, the conceptual model assumes that the hinterland water level $\:{h}_{down}$ equals the depth of critical flow $\:{d}_{c}=R\:({}_{}{h}_{up,t}-{}_{}{z}_{t})$, with $\:R=2/3$ in the base case (Sect. 2.3.3). The Root Mean Square Error (RMSE, Eq. 11) of the flood volume time series is computed for values of $\:R$ between $\:\left[\text{0,1}\right]$, comparing the conceptual model to the hydrodynamic model. The value of $\:R$ that leads to the lowest RMSE is considered the best value for that breach scenario. We also show the MAE (Eq. 12) in the results section. Both RMSE and MAE are computed only for time steps after breach initiation.

11 $$\:RMSE=\sqrt{\frac{1}{T}{\sum\:}_{t=1}^{T}{\left({V}_{t}-{\widehat{V}}_{t}\right)}^{2}}$$

12 $$\:MAE=\frac{1}{T}{\sum\:}_{t=1}^{T}\left|{V}_{t}-{\widehat{V}}_{t}\right|$$

Where:

- $\:T$ is the number of time steps in the simulation after the breach initiated
- $\:{V}_{t}$ and $\:{\widehat{V}}_{t}$ are the conceptual model’s flood volume and hydrodynamic model’s flood volume, respectively

In the hydrodynamic model simulations used for calibration, the dike breaches at the peak of the river discharge wave. This means that the dike breaches at different critical river water levels for the calibration scenarios: a relatively low water level for the T100 and a high water level for the T100,000 scenario. Therefore, two river-hinterland water level ratio $\:R$ values are found in the calibration for a breach location, corresponding to the critical river water levels of the T100 and T100,000 scenarios at that breach location. To be able to use the calibrated model with differing critical river water levels (e.g. T10,000 for the validation), a linear relationship between the $\:R$ values for the T100 and T100,000 scenarios is drawn based on the critical river water level. This linear relationship allows for finding suitable $\:R$ values in the validation scenarios, and its verification is shown in Sect. 3.1.

#### Confined breaches

For confined breaches, the value of $\:R$ requires calibration as well as the hinterland area parameter $\:A$, which governs the rise rate of the hinterland water level (Sect. 2.2). The ratio $\:R$ is varied between $\:\left[\text{0,1}\right]$ and the area $\:A$ is varied between $\:\left[10,\:10\:000\right]$ km^2^. The smaller $\:A$, the faster the breach drowns and the breach outflow becomes 0. For larger $\:A$, the breach drowns later. For the largest values of $\:A$, the breach behaves like an unconfined breach. This is because the hinterland water level never increases beyond the water depth of critical flow and the breach does not drown. Therefore, the range for finding $\:A$ for confined breaches is sufficiently large. Also for the confined breaches, linear relationships between the $\:R$ values and $\:A$ values are drawn based on the critical river water level for breach initiation of the calibration scenarios.

### Validation

The conceptual model is validated per breach location through additional simulations of different discharge waves (Table 1). This is to test the conceptual model’s applicability for a real-time flood forecasting system, so the characteristics of the incoming discharge wave can vary from event to event. We test discharge waves with different return periods (T1,000 and T10,000), a discharge wave with a broader shape (peak at T10,000) and one with two peaks (peak at T10,000) (Table 1). The dikes breach at the peak of the discharge wave events, which is the same time step in both the conceptual and hydrodynamic models.

### Application for uncertainty analysis

The application of the conceptual model is found in time-sensitive situations, such as an approaching extreme river discharge wave. The Dutch and German water authorities provide forecasted river discharges, generated through hydrological models based on weather predictions (e.g. Van Verseveld et al. 2024). However, uncertainties regarding the dike strength mean that it is unknown at which water level a dike may breach for such a forecasted discharge wave. Whereas failure due to wave overtopping and overflow is fairly certain to happen when the river water level reaches towards the dike crest, failure due to piping or macro-instability could happen at lower water levels. The conceptual model can account for this by computing an ensemble forecast of possible dike breach outflow hydrographs, with which an estimate of possible flood scenarios can be evaluated.

Uncertainty in breaching moments of dikes is often quantified using fragility curves for a specific failure mechanism (e.g. Vorogushyn et al. 2009; Bomers et al. 2019). These express the probability that a dike fails if the river reaches up to a particular river water level. In the study area of this research, fragility curves are available for each of the Dutch dike sections and specified for piping, macro-instability and overtopping (which includes both overflow and wave-overtopping). These failure mechanisms are often considered most relevant for riverine dikes (De Bruijn et al. 2014; Diermanse et al. 2015).

For each simulation in the ensemble forecast, the three fragility curves are weighted equally, so one critical water level for each failure mechanism is sampled. Similar to Diermanse et al. (2015), the lowest value of the three is then used as the critical water level for that simulation. If the river water level reaches this critical level, the conceptual model initiates the breach. Through repeated sampling and selection of the lowest value, a set of critical water levels is created that envelopes the range of possible breaching moments for a dike section with its specific fragility curves.

The conceptual model is then used to simulate the breach outflows for a particular river discharge wave, given this range of critical water levels. To show the application of the conceptual model in such a scenario, the T10,000 GRADE-standard discharge wave (Sect. 2.5) is analysed at the breach location at Loo (Fig. 1). The results are shown in Sect. 3.3.

## Results

### Calibration

The calibration is set up to find the water level ratio $\:R$ for various critical river water levels. Based on the calibrated values for these water levels, a trend line is fit through these points. With this trend line, appropriate values for $\:R$ can be found for flood events with different critical water levels in the validation phase (Sect. 3.2) and application (Sect. 3.3).

**Fig. 4:**
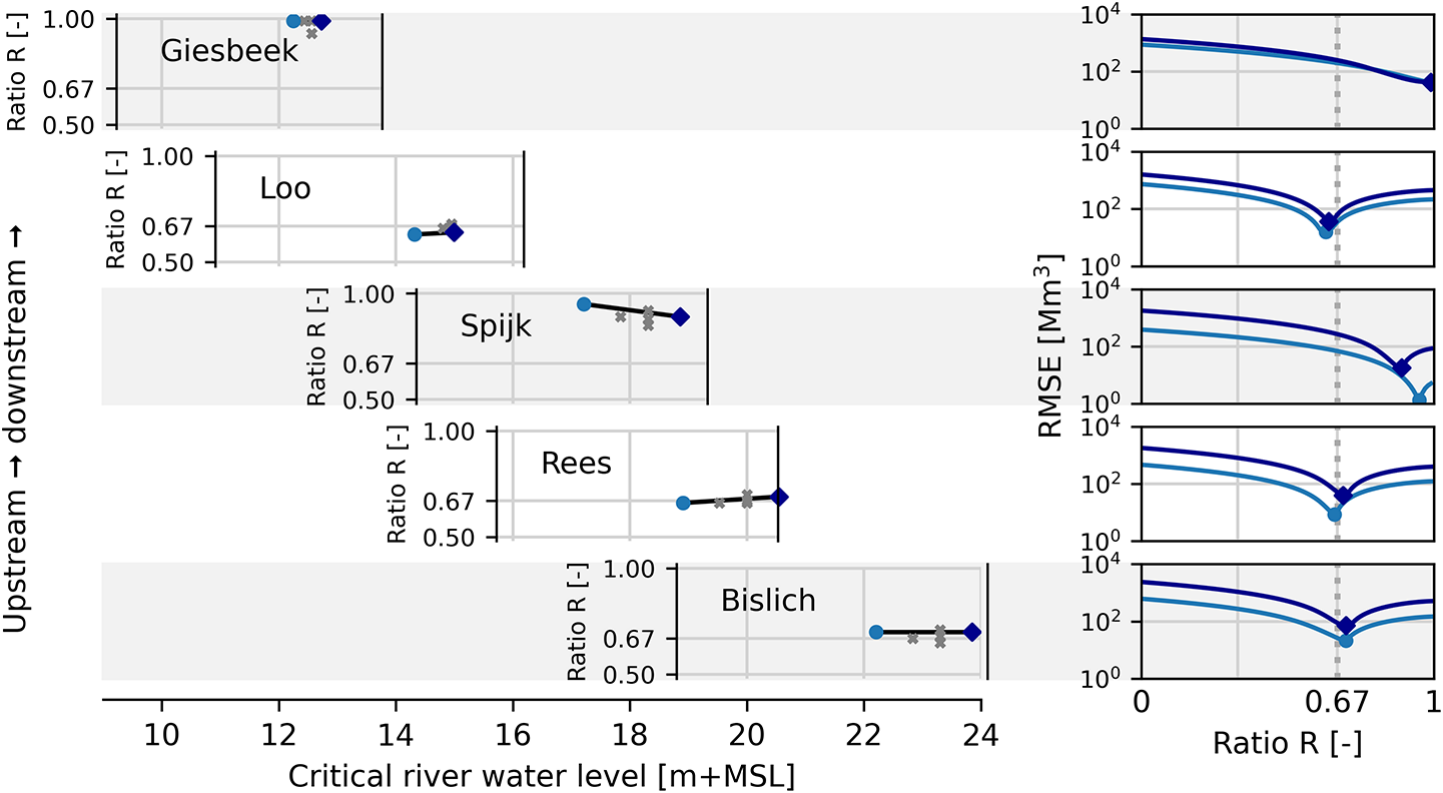
Calibration of the river-hinterland water level ratio $\:R$ for various flood events at the breach locations of Giesbeek, Loo, Spijk, Rees and Bislich, with river water level expressed above mean sea level. **Left**: Calibration values of $\:R$ against the critical river water level for the T100 (•) and T100,000 (♦) scenarios, with the trend line through the data points. The grey data points (×) show the ideal $\:R$ value for the validation scenarios determined by re-calibration. **Right**: RMSE of the flood volume result for the range of possible $\:R$ values for the T100 (•) and T100,000 (♦) calibration scenarios

Figure 4 shows the calibrated ratio $\:R$ values for five breach locations and for different flood events per location, if we assume that each breach location is unconfined (through infinitely large hinterland area values). The graphs in the right panels show how changing values of $\:R$ result in varying values of the objective function, i.e. the RMSE of the flood volume time series. For three of the five breach locations (Loo, Rees and Bislich, see also Fig. 1), the best values of $\:R$ with the lowest RMSE are around 0.67, which is the value for idealized unconfined breach locations. This means that these locations are properly characterized by the unconfined breach type.

In the left panels of Fig. 4, the $\:R$ values of the T100 (•) and T100,000 (♦) calibration flood events are plotted against the critical river water level. A linear $\:R$-trend line is shown connecting the two points. For scenarios with different critical river water levels (e.g. T1,000 and T10,000), the value of $\:R$ obtained from this linear trend line is used in the validation phase of Sect. 3.2. In Fig. 4, we also show the ideal values of $\:R$ for these validation scenarios as grey data points, if the model were calibrated specifically to these scenarios. This indicates that the ideal values of $\:R$ are not far removed from the values obtained through the $\:R$-trend line from the calibration procedure. The difference in $\:R$ values for the validation scenarios is at most 0.05, so we advise users of the model to adhere to a ± 0.05 uncertainty range in the $\:R$ values.

The breach location at Spijk (Fig. 1) achieves an optimum in the calibration for values of $\:R$ around 0.8–0.9, significantly higher than 0.67 (Fig. 4). This indicates that the characterization of this breach as the unconfined type is less valid. This can be caused by the hinterland geometry, which results in flood water from this breach location entering a separate dike-ringed area of approximately 40 km^2^. It accumulates here and eventually affects the breach outflow, though the effect is not very strong. Therefore, the increased values of $\:R$ found in the calibration mean that the conceptual model requires a reduced breach growth to match the hydrodynamic model’s reduced breach outflow. However, this breach location cannot be classified as a confined breach location, since a well-fitting optimum is found in the calibration. We show in Sect. 3.2 the results for this breach location under both the unconfined and confined breach methodologies.

The breach location at Giesbeek (Fig. 1) is shown to achieve no optimum value in the calibration, with the lowest RMSE values achieved at the maximum $\:R$-value of 1 (Fig. 4). This means that under the unconfined methodology, the hinterland water level should be almost equal to the river water level for the breach location. Therefore, this breach location cannot be properly characterized by the unconfined breach type. This can be explained by the topography of the area, as the breach is located in the lowest-lying point of the hinterland, meaning that flood water is not free to flow away unhindered. Therefore, this breach location is classified as a confined breach location.

**Fig. 5:**
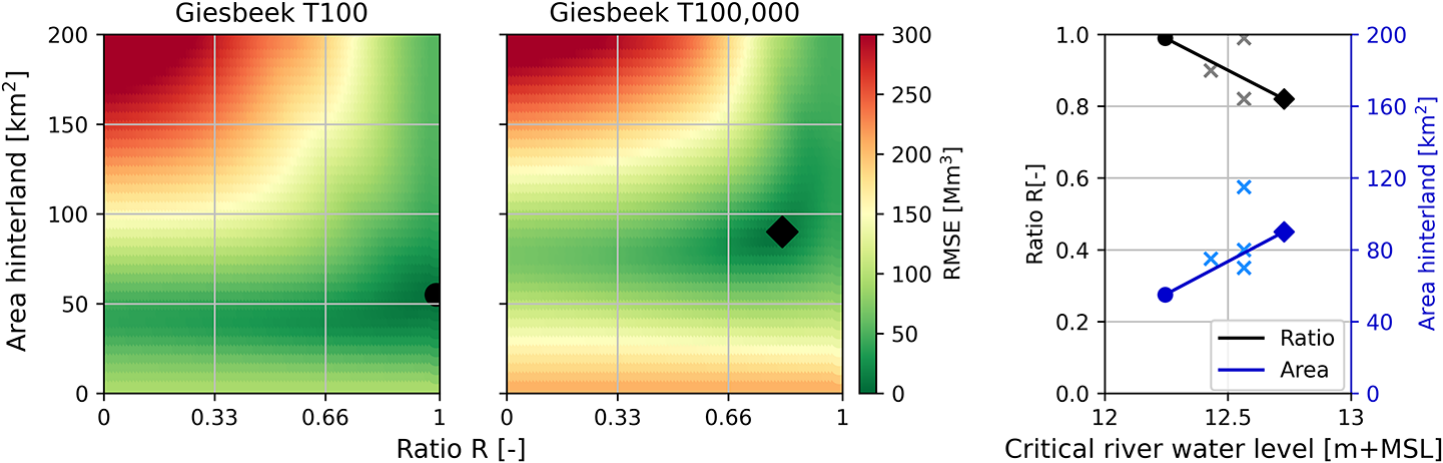
Two-dimensional calibration of the conceptual model parameters (river-hinterland water level ratio $\:R$ and area of the hinterland $\:A$) at the breach location Giesbeek. **Left**: Heat maps of RMSE of flood volume for T100 (•) and T100,000 (♦) scenarios, with black symbol indicating the best combination of $\:R$ and $\:A$. **Right**: Calibrated values of $\:R$ (black) and $\:A$ (blue) against the critical river water level for the T100 (•) and T100,000 (♦) scenarios, with the linear trend line through the data points. The grey and light blue data points (×) show the ideal $\:R$ and $\:A\:$values for the validation scenarios determined by re-calibration, respectively

The calibration of confined breach locations requires the area parameter $\:A$ (Sect. 2.3.4). Figure 5 shows for the Giesbeek breach location the RMSE of the model for various combinations of the river-hinterland water level ratio $\:R$ and the area of the hinterland $\:A$. Note that $\:A$ was varied between [10–10 000] km^2^, but we show the results in the range of [10–200] km^2^ where the optima were found. The most optimal value of $\:R$ is found to be between 0.8 and 1 for the two calibration scenarios, while the value for $\:A$ is around 50–90 km^2^ depending on the critical river water level. The validation scenarios are shown in Fig. 5 with lighter colours, showing the difference between the ideal value and the linear relationship obtained through the calibration. The average error is ± 0.05 for the $\:R$-value and ± 15 km^2^ for the $\:A$-value.

The trend lines of the calibration parameters show a decreasing trend in the ratio value $\:R$ and an increasing trend for the area of the hinterland $\:A$, for increasing critical river water levels. These trends can be explained by the fact that for higher return period discharge waves, more water enters the hinterland. In the conceptual model, this is reflected through lowering values of $\:R$ to enforce more breach growth and breach discharge, and through increasing values of $\:A$ to store more flood volume.

### Validation

With the calibration parameter trend lines, fitting values of $\:R$ and $\:A$ for the validation scenarios are found using the threshold critical river water level for breach initiation. In the next section, we show the results of these fitted values of $\:R$ and $\:A$, alongside the results of the most optimal values of $\:R$ and $\:A\:$(Figs. 4 and 5). The optimal values for the validation scenarios are not far removed from the found calibration parameter relationships, with an error margin of ± 0.05 for $\:R$ and of ± 15 km^2^ for $\:A$.

#### Unconfined breach locations

At breach location Loo, the conceptual model performs well for the validation discharge waves (Table 2). The volume curves are quite accurately matched by the conceptual model, and the deviations mostly fall within the ± 0.05 $\:R$-value error margin. The RMSE of the breach outflow and flood volume is shown to be between 10 and 15% away from the peak values.

**Table 2:**
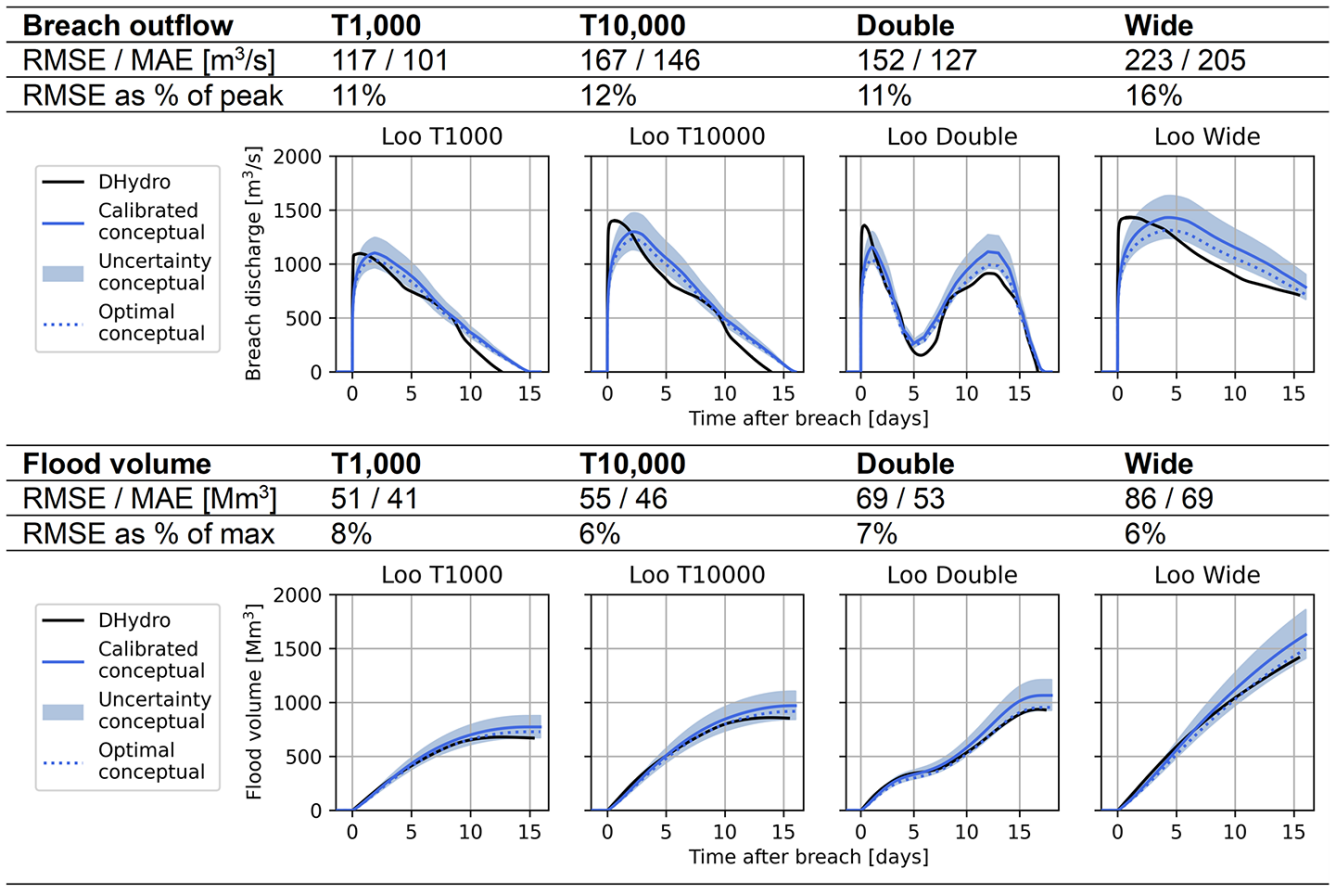
Validation scenarios for breach location Loo, showing the performance of the conceptual model compared to the hydrodynamic DHydro model. “Calibrated conceptual” refers to the model created by the calibration procedure, “optimal conceptual” shows the performance if the conceptual model was specifically calibrated for each validation event

The hydrodynamic model reaches the peak breach outflow almost immediately after the breach occurs, while the conceptual model reaches its peak breach outflow about two days later. A reason for this is that the river water level of the hydrodynamic model drops slightly due to the breach outflow, and lower river water levels result in lower breach outflows. Therefore the highest water level difference and therefore the highest breach outflow only occurs at the very beginning of the breach. The conceptual model does not take into account this effect, thus its peak breach outflow is achieved some time later when the breach has widened more.

For the German breach locations at Bislich and Rees (Fig. 1), the behaviour of the conceptual model is very similar. In this section we show the results of Bislich (Table 3; error around 15%), which shows less accurate performance than Rees (Appendix–Table 6, error around 10%).

**Table 3:**
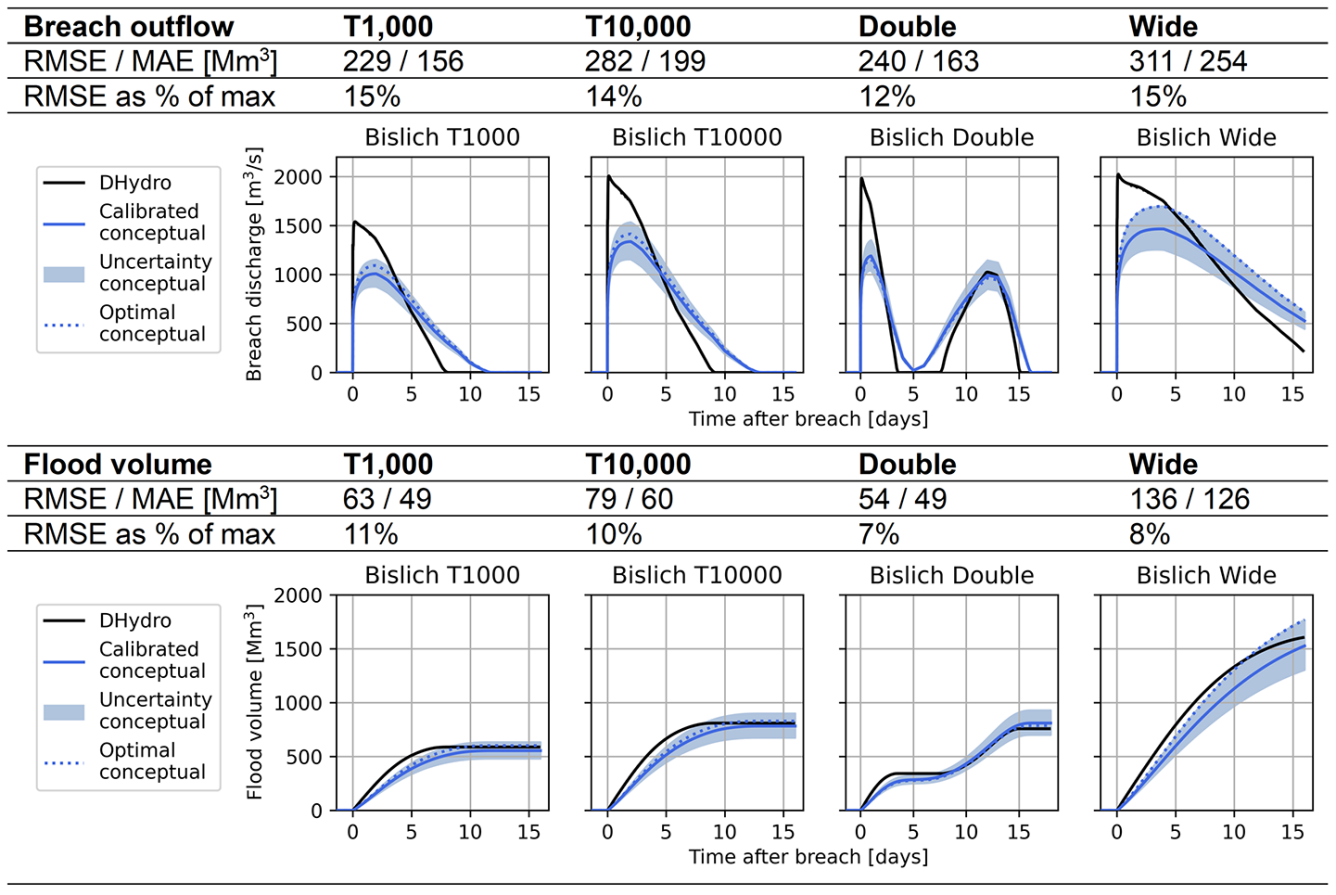
Validation scenarios for breach location Bislich, showing the performance of the conceptual model compared to the hydrodynamic model. “Calibrated conceptual” refers to the model created by the calibration procedure, “optimal conceptual” shows the performance if the conceptual model was specifically calibrated for each validation event

The conceptual model underestimates the initial peak of the breach outflow at the Bislich location, and shows a more slowly decreasing breach outflow compared to the hydrodynamic model (Table 3). The final flood volume is matched well, but approximately two days later for the T1000 and T10,000 scenarios. Note that for the double-peak discharge wave, the hydrodynamic model breach outflow becomes zero between the two peaks. This is because the river water level recedes to equal the hinterland water level shortly after the first peak. The conceptual model breach outflow reaches to zero for a shorter time period than the hydrodynamic model. This is caused by the unconfined breach’s free-flow, due to which the hinterland flood water level does not increase to levels that affect the breach outflow computation.

The breach location at Spijk (Fig. 1) required values of the river-hinterland water level ratio $\:R$ higher than the unconfined breach type base value of 0.67 in the calibration (Fig. 4). The flood water enters a separate dike-ringed area, which results in the unconfined breach type being less valid at this location. In Table 4, we show the results for the Spijk location using both the unconfined and the confined breach calibration methodologies. The results are very similar, indicating that the obstruction of the breach outflow is not strong enough to require specific calibration with the area parameter $\:A$. Calibration using the river-hinterland water level ratio $\:R$ proves to be sufficient for finding a good result. Therefore, we advise to start calibration for any scenario with only the ratio $\:R$, and seek if an optimum in the calibration is achieved. For breach locations where no optimum is found, we recommend to introduce the second calibration parameter $\:A.$ This is the case for the confined breach location at Giesbeek, discussed in the next section.

**Table 4:**
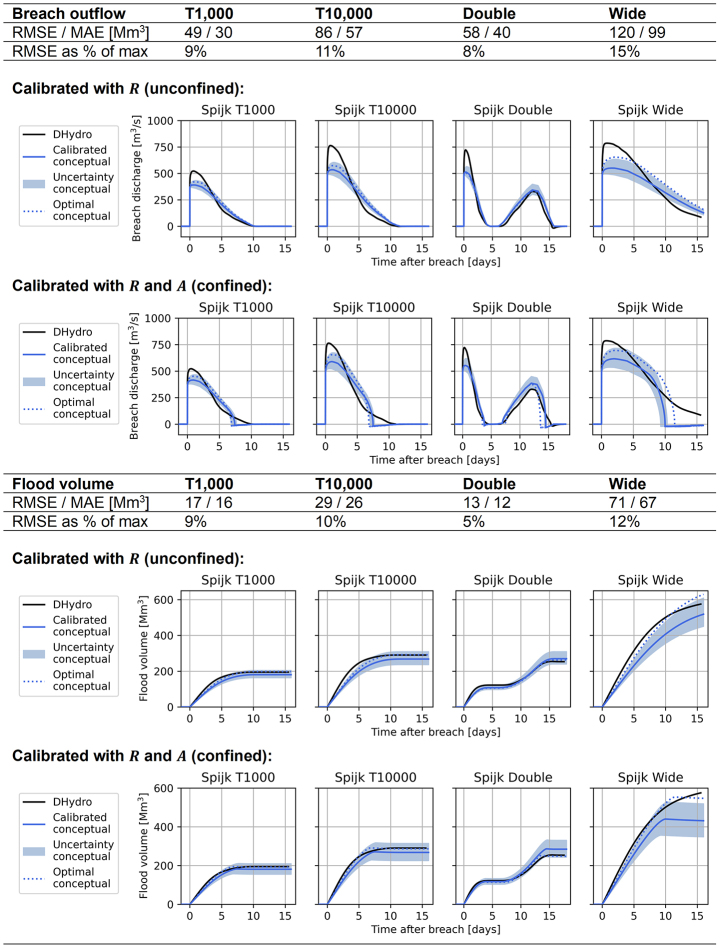
Validation scenarios for breach location Spijk, showing the performance of the conceptual model compared to the hydrodynamic model. The values indicate the performance for the unconfined calibration methodology (using only $\:R$), while the plots show the results of both the unconfined and confined calibration methodologies. “Calibrated conceptual” refers to the model created by the calibration procedure, “optimal conceptual” shows the performance if the conceptual model was specifically calibrated for each validation event

#### Confined breach location

At the confined breach location of Giesbeek (Fig. 1), the breach outflow quickly reduces due to the effects of the hinterland water level. This is caused by the breach’s location in a low point of the hinterland. To reflect this in the conceptual model, the area parameter $\:A$ determines when the conceptual model switches from the unsubmerged broad-crested weir equation to the submerged version (Sect. 2.3, Eqs. 8 and 10 respectively). This moment is visible in the breach outflow hydrographs of Table 5, where the breach outflow decreases sharply. Shortly after, the breach outflow also becomes slightly negative, as the hinterland water level exceeds the river water level and water flows back into the river.

**Table 5:**
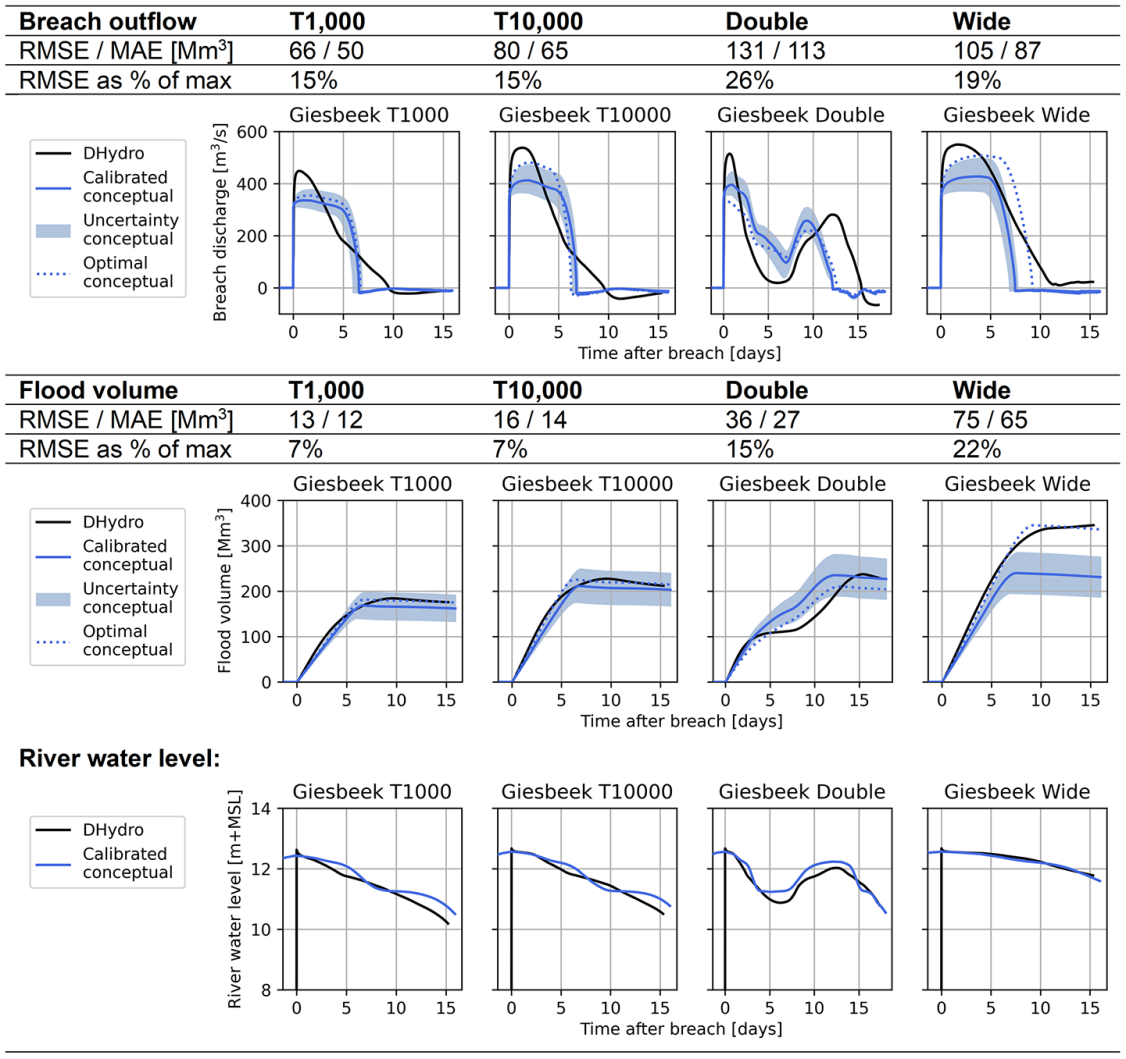
Validation scenarios for breach location Giesbeek, showing the performance of the conceptual model compared to the hydrodynamic model. Additionally, the river water level comparison is shown for the different scenarios. “Calibrated conceptual” refers to the model created by the calibration procedure, “optimal conceptual” shows the performance if the conceptual model was specifically calibrated for each validation event

The conceptual model underestimates the peak of the breach outflow and is not able to capture the behaviour of a gently decreasing breach outflow (Table 5). Possibly, this is caused by the switching point between the unsubmerged and submerged broad-crested weir equation being abrupt; the conceptual model considers no effects of the hinterland water level on the breach outflow until it exceeds the depth of critical flow $\:{d}_{c}$ (Eq. 6). Another cause for worse performance is the stage-discharge relationship (bottom panel Table 5), which shows a sharper and non-gradual decrease of water levels than the hydrodynamic model. Especially for the double-peak river discharge wave scenario, the resulting fluctuating over- and underpredictions in the river water level translate to fluctuating over- and underpredictions of the breach outflow. Finally, for the wide discharge wave the volume is underestimated: the most optimal conceptual model is outside the range of uncertainty of the calibrated conceptual model. This is because this long flood event requires the conceptual model to have a larger hinterland area parameter $\:A$ to store more volume. The uncertainty range of ± 15 km^2^ on the area parameter meant to balance for these deviations is the average and not sufficient for this scenario. The most optimal value for $\:A$ deviates 35 km^2^ from the calibrated relationship (see Fig. 5).

#### Aggregated comparison

**Fig. 6:**
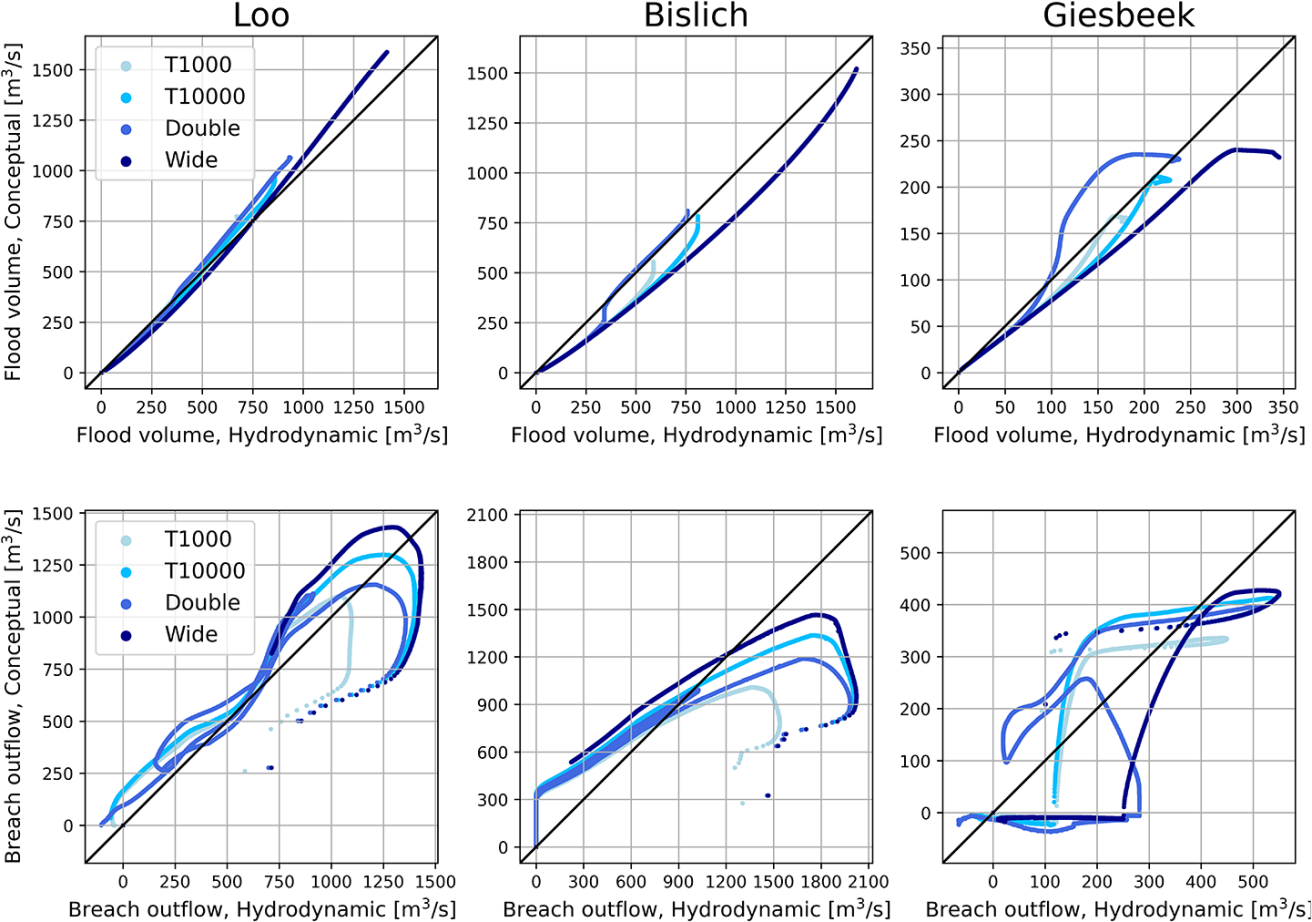
Scatter plots of flood volume (upper row) and breach discharge (lower row) of conceptual model against hydrodynamic model, for breach locations Loo, Bislich and Giesbeek. Black $\:y=x\:$line shows perfect prediction

Scatter plots show the conceptual model performance compared to the hydrodynamic model for all validation scenarios for breach locations Loo, Bislich and Giesbeek (Fig. 6). Locations Rees and Spijk are shown in Appendix–Figure 11. For the flood volumes, the conceptual model agrees reasonably well with the hydrodynamic model. Especially the breach location at Loo performs well, with the data points consistently near the $\:y=x$ line that indicates a perfect prediction. The breach location at Bislich shows an underestimation of the flood volume by the conceptual model, caused by the underestimation of the outflow in the first days of the flood. The confined breach location of Giesbeek shows the largest discrepancies between the two models. Contributing factors are the sudden switch from the submerged to unsubmerged breach discharge equation, as well as the poor fit of the stage-discharge relationship causing significant differences in the river water level between the conceptual and hydrodynamic models, and consequently the breach outflow (Sect. 3.2.2).

Regarding the breach outflow of the unconfined breaches, the data points are mostly concentrated along the $\:y=x$ line. For the higher breach outflows, the conceptual model shows larger deviations from the hydrodynamic model results. This corresponds to the high initial peak in the breach outflow, which was found to be underestimated by the conceptual model (Sect. 3.2.1). A reason for this is that the calibration was conducted on the RMSE of the flood volume, so errors in the breach outflow were only indirectly affected during calibration. Another reason for the deviations in the breach outflow results is that the breaching process is highly dynamic, while the conceptual model aims to capture it in only a few static parameters. We include a discussion on this in Sect. 4.2.

### Application for uncertainty analysis

**Fig. 7:**
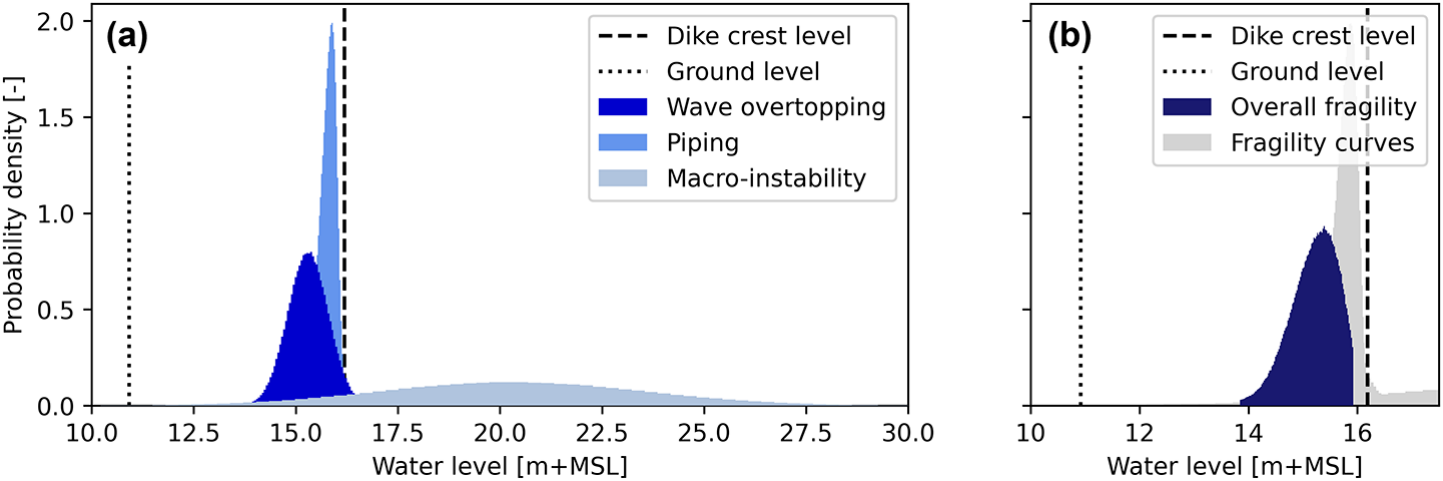
(**a**) Probability density plots of the fragility curves for piping, macro-instability and combined wave-overtopping plus overflow, for the breach location of Loo. (**b**) Resulting 95% confidence interval of the fragility curves after repeated sampling from the three separate fragility curves and taking the minimum

The validated conceptual model is applied to probabilistically study how the breach outflow is affected by the uncertainty around the critical river water level, using fragility curves for the breach location at Loo (Fig. 7a). Repeated sampling of the three fragility curves and taking the minimum of each set resulted in an overall set of critical water levels. This set is mainly dominated by the piping failure mechanism. However, some very low critical water levels sampled from the macro-instability failure mechanism mean that the dike breaches at river water levels barely above ground level and far below the dike crest level. We continue the analysis with the 95% confidence interval of the samples, to exclude these extreme outliers (Fig. 7b).

**Fig. 8:**
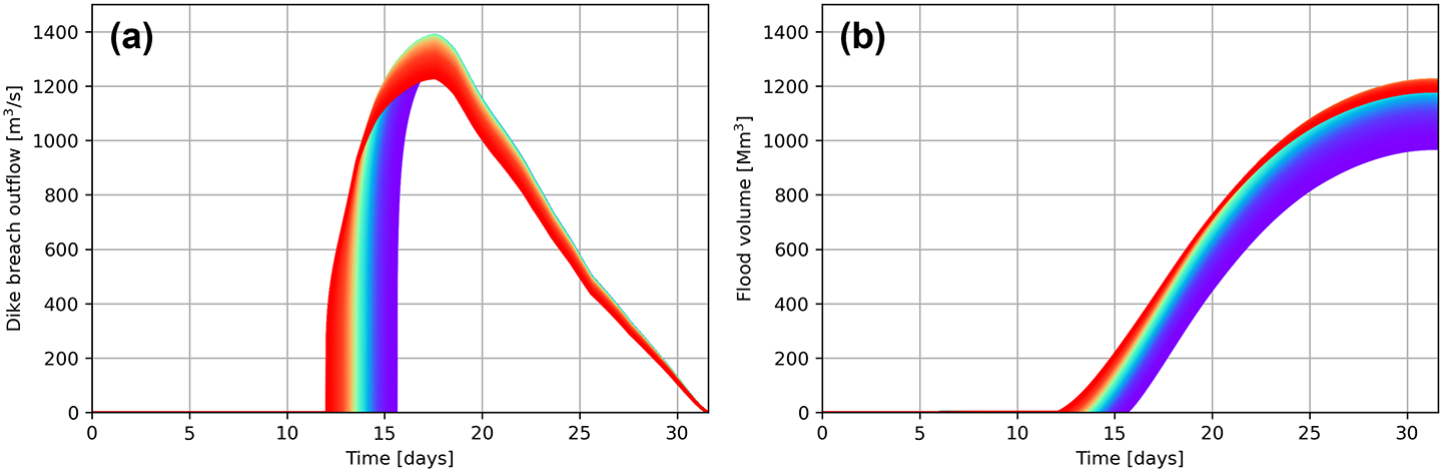
(**a**) Probabilistic breach outflow for varying breaching moments. Earlier breaches are coloured red, later breaches tend via green towards purple. (**b**) Probabilistic flood volume for varying breaching moments

The conceptual model runs 9500 scenarios of varying critical water levels with the T10,000 river discharge wave in 2 min (simulation time of 31 days, time step of 10 min). The spread of breaching moments for this Dutch standard-shape discharge wave is approximately four days. Figure 8 shows the resulting uncertainty in breach outflow and flood volume. As expected, earlier breaches insert more volume into the hinterland due to the longer opening time of the breach: about 20% more than late breaches.

**Fig. 9:**
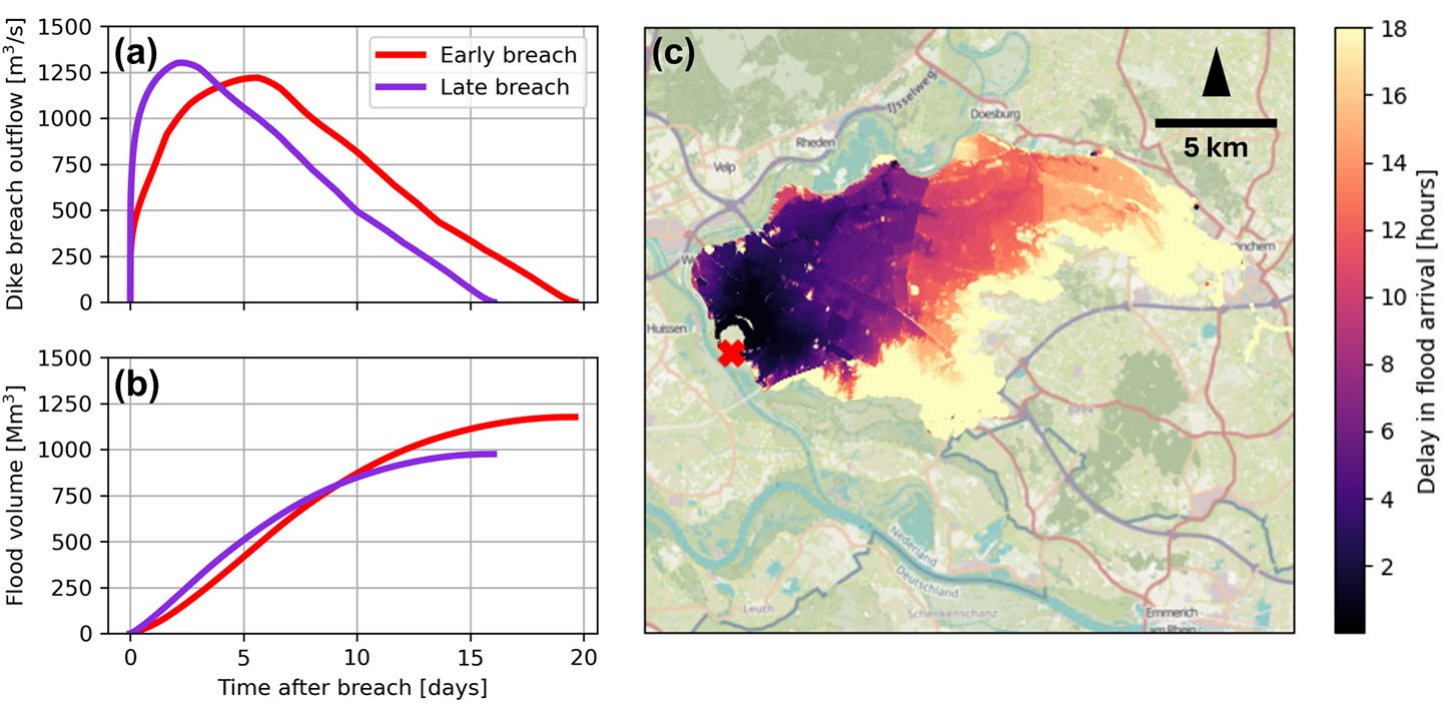
(**a**) Dike breach discharge and (**b**) flood volume over time for scenarios in which the breach occurs at a lower river water level (“early breach”) and at a high river water level (“late breach”). (**c**) Further away from the breach (marked with red X) a significant delay in the arrival times is visible for the scenario “early breach” compared to the scenario “late breach”

The conceptual model does not provide 2D flood propagation results, so the hinterland flooding was simulated using the hydrodynamic model to illustrate the significance of the spread in outflow hydrographs. The earliest and latest outflow hydrographs produced by the conceptual model were used as its input (Figs. 8a and 9a). The difference in arrival times is shown as the delay of the early breach scenario compared to the late breach scenario (Fig. 9c). Due to the lower river water level at an early breach, the initial breach outflow is lower and the hinterland floods slower. The early breach scenario shows a delay in arrival time of 12 to 18 h for the furthest sections of the hinterland. It takes about 40 h for the flood water to flow that distance from the breach, so a delay of 12–18 h is quite significant for evacuation success rates and planning of possible evacuation strategies. This shows the value of the conceptual model in that it enables such uncertainty analyses using the quick estimation of breach outflows. With the large uncertainties surrounding the processes involved in dike breaches, research into reducing these uncertainties is vital for improving dike breach flood prediction.

## Discussion

In this research, a conceptual model to estimate dike breach outflow is proposed and assessed. We discuss the applicability of the model, limitations of its setup and how the model fits in a full framework for dike-breach flood modelling using surrogate models.

### Applicability

The developed conceptual model can estimate the dike breach outflow quickly and within reasonable accuracy of a hydrodynamic model, for varying river discharge wave shapes and peaks. This allows decision makers to explore the effects of uncertainties surrounding the dike breaching process during situations when limited simulation time is available.

The model does not require many data in order to function for another breach location or for another river system. First, a stage-discharge relationship or other approach to find the river water level is needed. Second, the Verheij-van der Knaap breach growth formula contains two parameters that change if the dike has a sand core or a clay core. Then, a river discharge wave of interest and a critical water level of interest need to be defined.

The final two input parameters are the calibration parameters of the model: the hinterland-water level ratio and, if needed, the area of the hinterland. We find that for unconfined breach locations where the outflow is not hindered by local topography, an initial estimate of the calibration parameter around 0.67 usually provides a sufficiently accurate range of outflow hydrographs. Therefore, by assessing the general local terrain behind a breach location, we consider it possible to use the model in situations where no hydrodynamic model is available. This approach is valid in river deltas, where the terrain is flat behind the breached dike, and not in river valleys, where the terrain slopes upward behind the breached dike. If the assessment of the local terrain shows that the breach is confined by topography changes or by an upward sloping hinterland, the approach requires two hydrodynamic model simulations to calibrate the parameters for the strength of the hinterland effect.

### Limitations

A limitation of the current approach is the static nature of the river water level implementation. The lowering of the river water levels due to the breach outflow is not taken into account, which results in a smaller head difference across the breach and thus lower breach outflow. However, initial tests with a forced water level reduction indicated only a small volume change of 2% in the conceptual model results. Additionally, the current case study is around bifurcating rivers, which means that a breach in one of the branches can draw more river discharge towards that branch (Bomers et al. 2019; Gensen et al. 2020), which may negate the effect of the water level reduction slightly. A hydrodynamic river model (1D or 2D) is required to fully take these dynamics into account, but this would have an impact on the simulation time of the model.

As presented in Sect. 3, we find that for unconfined breach locations the approach with the stage-discharge relationship works well. For more complex hinterland interactions the approach requires more specific calibration. A similar result is found by Kamrath et al. (2006), who employ a broad-crested weir discharge equation and a 1D river model to find a fast estimate of dike breach outflow. While their research does not include dynamic breach growth, instead having a constant breach width, they also find that the inclusion of backwater effects requires more reliable relationships to be established, before being properly implemented in a real-time flood forecasting model.

Thirdly, the empirical Verheij-Van der Knaap breach growth equation is implemented, but the computed breach width is not a performance indicator of the conceptual model. Rather, the breach width effectively functions as a calibration value to account for detailed flow processes that take place in and around the breach. Modelling the breach width accurately was not a goal of this research; the flood volume and breach outflow are more important in order to eventually model the spread of flood water in the hinterland, and no historical data of breaches in this study area are available for validation. Additionally, different breach growth equations exist (e.g. Visser 1998; Van Damme 2020), some of which include more physical parameters such as the dike soil composition. Although these breach growth methods are not often implemented in hydrodynamic models, the conceptual model is theoretically able to incorporate such different equations. Here, we compare our conceptual model to a hydrodynamic model that utilizes the Verheij-Van der Knaap breach growth equation, so we cannot verify if the conceptual model improves with respect to reality through the use of other breach growth models. Additionally, the added complexity makes the conceptual model less appealing for use in time-sensitive applications.

Similarly, different breach outflow equations can be incorporated into the model. Specifically, we would like to discuss the use of the side weir equation, due to its conceptual similarity to dike breaches. We have evaluated the use of the side weir equation in the conceptual model and found that it strongly depends on the discharge coefficient that is computed for the situation. Many authors have experimentally derived equations for this coefficient, which often rely on the Froude number upstream of the side weir (see for example Lee et al. 2019; Rifai et al. 2018; Schmitz et al. 2024). The stage-discharge relationships used to compute the river water level for our conceptual model do not deliver the flow velocity needed to compute the Froude number. Therefore, the discharge coefficient of the side weir equation would require additional calibration of the conceptual model, possibly leading to overfitting. To enable a more robust use of the side weir equation, the coupling to a 1D river model can be a solution, as it offers the additional flow information to compute side weir discharge coefficients at the cost of computation time.

Finally, the outflow during a dike breach is inherently linked to the processes that happen in the hinterland. In this research, calibration parameters were implemented that remain static throughout a simulation. To compensate for the underestimation of the initial peak breach outflow, we conducted tests with time-varying values of the calibration parameter $\:R$. However, this lead to a more demanding calibration procedure and to overfitting for each specific scenario. Therefore, we proceeded with the static $\:R$-value, which was found to be applicable in our evaluated set of circumstances. However, at confined breach locations where the hinterland water level has a strong effect on the breach growth and breach outflow, the simple calibration rules of this research are less applicable. In the Giesbeek case presented in Sect. 3.2, the calibrated value for the hinterland water level ($\:R\sim1$) means that the hinterland water level should equal the river water level; i.e. there is no breach growth besides the breach width initiation. This is not very realistic, and it means that more attention should be given to the conceptualization of hinterland water levels, or the surrogate modelling of hinterland water level (Sect. 4.3).

### Surrogate dike-breach modelling

**Fig. 10:**
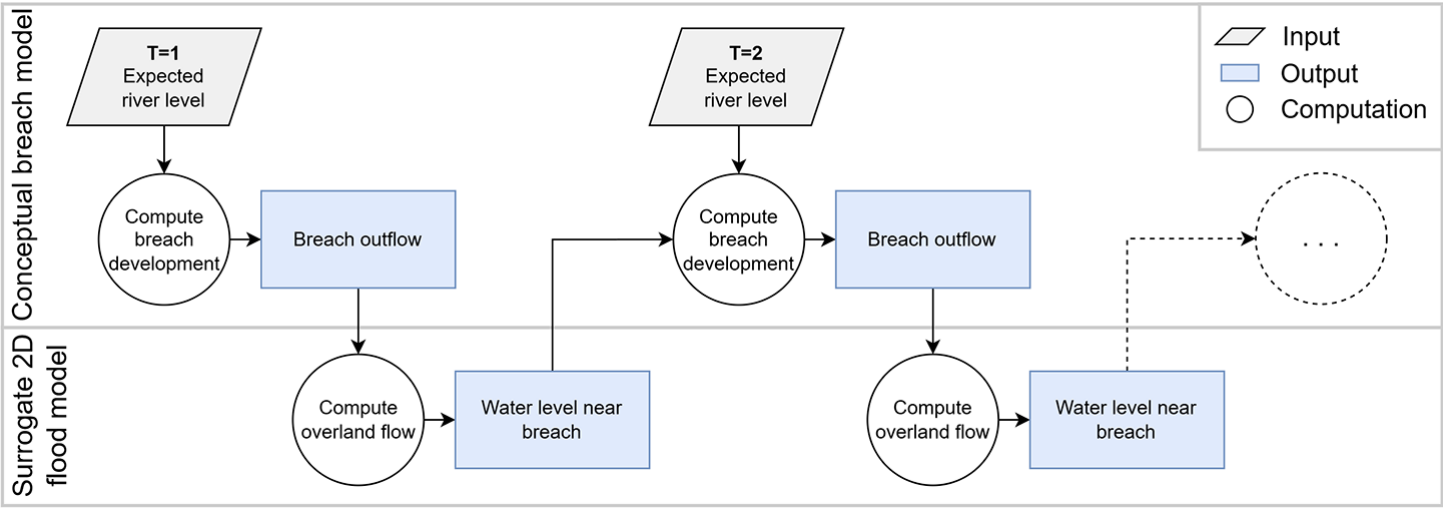
Setup of a modelling system for dike breaches, with a conceptual model for breach outflow linked to a surrogate 2D overland flow model

Full dike breach hazard assessment framework such as in Vorogushyn et al. (2011) and Maranzoni et al. (2022) have so far relied on computationally expensive hydrodynamic models: a 1D river model connected via a breaching module to a 2D hinterland model. To fully model the dike breaching system using fast surrogate models instead, approaches for breach outflow modelling and for 2D flood modelling need to be coupled. The conceptual model presented in this study is a surrogate of the 1D river model as well as the breach module of these hazard assessment frameworks. In future work, a 2D overland flow module can be captured by a surrogate model too, and coupled to the conceptual breach outflow model explored here. Figure 10 shows the possible setup of such a model, which would be able to better reflect the local hinterland processes that have made the confined breach locations in this research a challenge. With such a setup, the calibration parameter for the hinterland area $\:A$ used in this research would not be required.

Two possible directions exist for 2D surrogate models that can be coupled to the conceptual model developed in this study. First, machine learning techniques that are able to take a volume of water as input and spread it across a topology can be used (e.g. Bentivoglio et al. 2023; Besseling et al. 2024). Another option is to use conceptual models such as the Rapid Flood Spreading Method (RFSM; Lhomme et al. 2008), or the Height Above Nearest Drainage (HAND; Nobre et al. 2011) model for the hinterland, to distribute volumes across the topography and return water levels near the breach. Such approaches could offer more realistic modelling of the backwater effects for confined breach locations compared to the methodology offered in this study, leading to a more widely applicable surrogate model.

## Conclusion

In this study, we developed a conceptual model that captures the dike breach processes required for estimating the breach outflow of large-scale dike breach flood events. This model is able to compute the breach outflow in less than a second, taking forecasted river discharge as its input. Such rapid modelling allows scenario analysis and probabilistic analysis, which can provide important information to decision makers during an emergency. In the case of dike breach modelling, obtaining an indication of the probabilistic outflow hydrograph is important, because it dictates the flood volume entering the hinterland and corresponding casualties and damages.

The conceptual model is based on a river’s stage-discharge relationship, the empirical Verheij-van der Knaap breach growth formula, the broad-crested weir equation for the outflow, and a 0D hinterland characterization. It performs well for breach locations where water is able to flow away unhindered into the hinterland, achieving an error of 10 to 15% on the breach outflow and flood volume estimation compared to the hydrodynamic model, for varying discharge wave shapes and peak discharges. For breach locations where flow into the hinterland is more confined, the conceptual model requires more specific calibration per scenario and is less suitable for consistent estimation of the breach outflow to any new scenario.

**Appendix: additional breach location r** **esults**

**Table 6:**
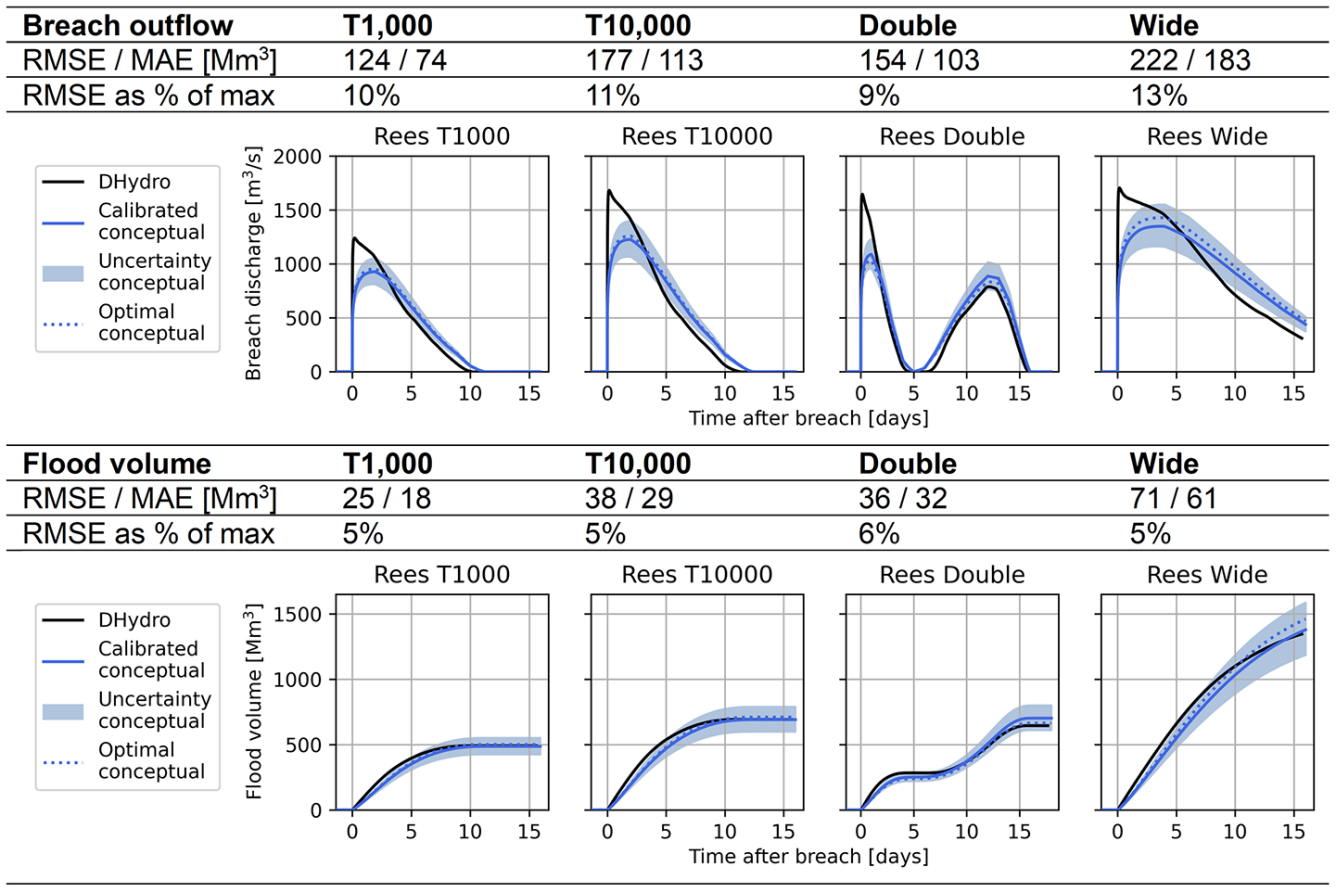
Validation scenarios for breach location rees, showing the performance of the conceptual model compared to the hydrodynamic model. “Calibrated conceptual” refers to the model created by the calibration procedure, “optimal conceptual” shows the performance if the conceptual model was specifically calibrated for each validation event

**Fig. 11:**
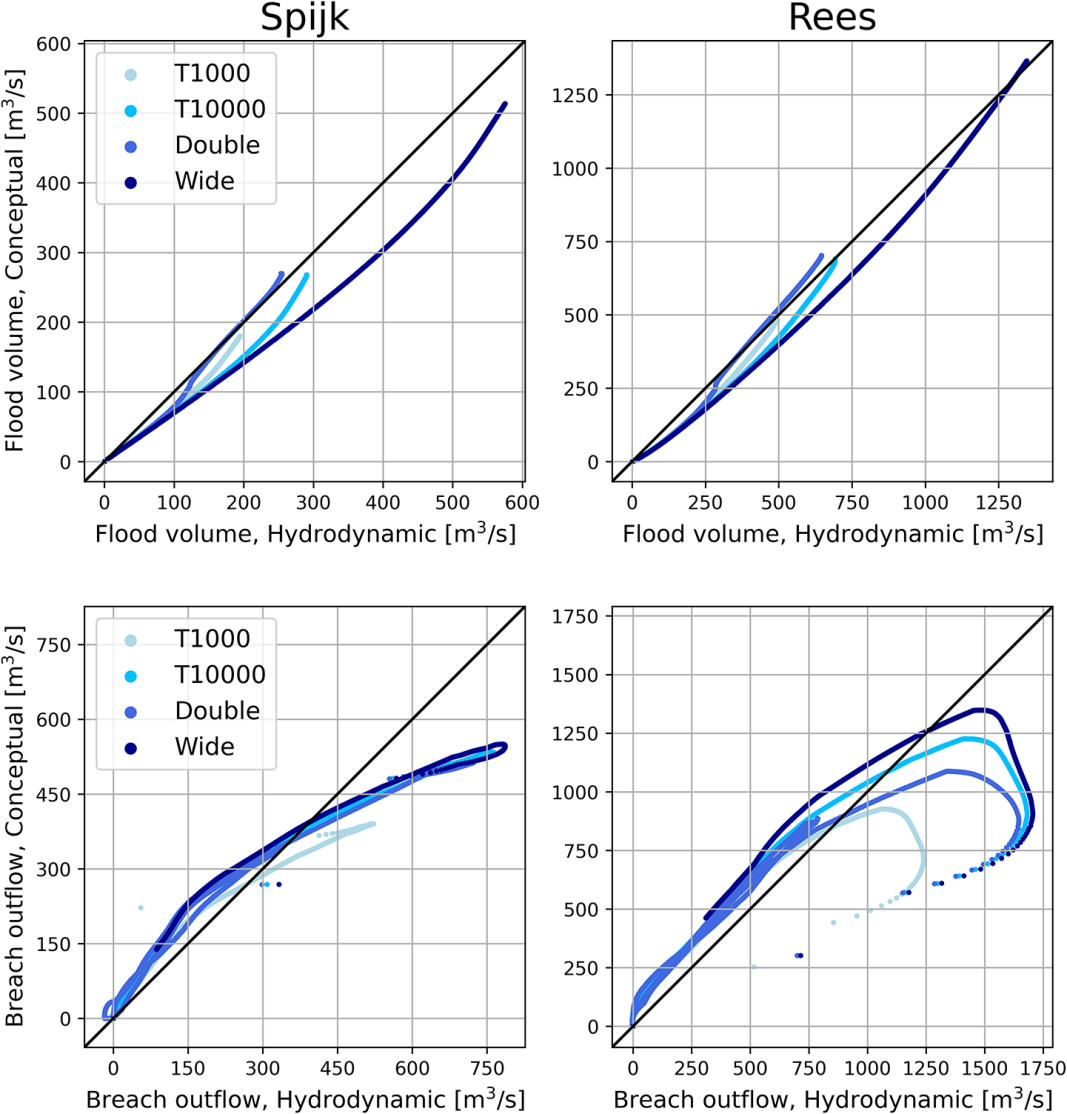
Scatter plots of flood volumes and breach outflows for breach locations Spijk and Rees
